# Genetic analysis of congenital and adult-onset lactose intolerance points to anti-inflammatory effects of dairy products

**DOI:** 10.1136/bmjnph-2024-001036

**Published:** 2025-07-03

**Authors:** Aytac Gul, Oliver F Ryder, Elham Alhathli, Alan Kelsall, Thomas Julian, Johnathan Cooper-Knock

**Affiliations:** 1 Hatay Mustafa Kemal University, Antakya, Turkey; 2 The University of Sheffield, Sheffield, UK; 3 The University of Manchester, Manchester, UK

**Keywords:** Food intolerances

## Abstract

**Objective:**

Dairy intake has been reported to be both pro-inflammatory and anti-inflammatory; this inconsistency has led to uncertainty in the field. We aimed to address this using genetic data to perform a causal analysis of the link between lactose intolerance, rheumatoid arthritis (RhA) and body mass index (BMI). Lactose intolerance necessarily leads to reduced dairy intake. RhA is an autoimmune disease, which has been linked to both increased and reduced dairy intake. Dairy intake has also been associated with elevated BMI, which is itself pro-inflammatory and is associated with increased risk of RhA.

**Methods:**

We separately considered congenital lactose intolerance, and adult-onset lactose intolerance where a prolonged period of dairy intake has already occurred. We combined Mendelian randomisation (MR) and rare genetic variant association testing to determine the relationship between genetic liability to lactose intolerance, risk of RhA and BMI. As a positive control to optimise instruments for measurement of lactose intolerance, we used the causal link between lactose intolerance and osteoporosis. Rare variant analysis was performed under a recessive model. Conditional analysis of the effect of lactose intolerance on the risk of RhA via BMI used multivariable MR.

**Results:**

We observed an opposite effect of congenital and adult-onset lactose intolerance on RhA risk. Congenital lactose intolerance increases the risk of RhA, but adult-onset lactose intolerance reduces the risk of RhA. The protective effect of adult-onset lactose intolerance on RhA is conditional on reduced BMI.

**Discussion:**

We conclude that dairy intake is anti-inflammatory, which explains why congenital lactose intolerance and lifetime avoidance of dairy foods increases the risk of RhA. However, adult-onset lactose intolerance can reduce the risk of RhA because of an association with reduced BMI, which is also anti-inflammatory.

**WHAT IS ALREADY KNOWN ON THIS TOPIC**

- Intake of dairy products is globally widespread, but it is controversial as to whether this is beneficial. For example, dairy intake has been reported to be both pro-inflammatory and anti-inflammatory in different studies; this includes in relation to the autoimmune disease rheumatoid arthritis (RhA).

**WHAT THIS STUDY ADDS**

- We have used genetic data to perform a causal analysis of the links between lactose intolerance, RhA and body mass index (BMI). Congenital lactose intolerance increases the risk of RhA, but adult-onset lactose intolerance reduces the risk of RhA. The protective effect of adult-onset lactose intolerance on RhA is conditional on reduced BMI.

**HOW THIS STUDY MIGHT AFFECT RESEARCH, PRACTICE OR POLICY**

- Our findings have translational implications and offer a mechanistic understanding of apparently contradictory findings in the literature. We conclude that dairy intake is likely to be anti-inflammatory, which explains why congenital lactose intolerance increases the risk of RhA. Dairy avoidance in adulthood has been posed as beneficial, but our work suggests that this may be indirectly mediated via reduction in BMI.

## Introduction

 Dairy intake is an important source of calories globally, but it has been reported to be both pro-inflammatory^1^ and anti-inflammatory^2^ in different studies. Indeed, dairy product consumption has been proposed as the major environmental factor underlying the set of chronic diseases which is common to industrialised societies.^1^ The fundamental issue is the difficulty in separating dairy intake from other associated factors which modify inflammation. As non-dairy substitutes become increasingly available, determining whether or not dairy products carry specific benefits or harms has significant translational implications.

Lactose intolerance necessarily results in reduced intake of dairy products. To be absorbed from the intestine, lactose, which is present within dairy products, must be hydrolysed by the enzyme lactase. Lactose intolerance consists of adverse gastrointestinal symptoms after consuming lactose-containing foods and beverages^3^ caused by incomplete lactose digestion due to deficiency of functional lactase. Lactose intolerance can be congenital as a result of homozygous or complex heterozygous mutations within lactase.^4^ However, even without genetic loss-of-function (LOF), levels of lactase reduce through life in a majority of individuals,^5^ and thus the majority of lactose intolerance actually presents in adulthood. Indeed globally, ~68% of the population is lactose intolerant to some degree, with varying prevalence across regions.^6^ In order to determine the effect of dairy product intake, we have used genetic liability to lactose intolerance as a natural experiment leading to reduced dairy intake.

The role of dairy intake and systemic inflammation is debated. A recent meta-analysis concluded that dairy products are likely to be anti-inflammatory except in relatively rare cases of milk allergy.^7^ Consistent with this, dairy products contain a number of anti-inflammatory lipids.^8^ In contrast, lactose-intolerant individuals tend to have higher levels of bifidobacteria and other bacteria within their gut microbiome^9^ that produce anti-inflammatory short-chain fatty acids from undigested lactose.^10^ Rheumatoid arthritis (RhA) is an autoimmune disease driven by inappropriate inflammation. RhA is relatively common with a prevalence of approximately 1%^11^; it is threefold more common in women who are relatively more predisposed to autoimmune diseases. It has been suggested that dairy products can trigger autoimmune diseases including RhA.^12^ However, other evidence points to a negative association between consumption of dairy products and risk of RhA.^13^ In our study of intake of dairy products, we have taken genetic liability to RhA as a proxy for harmful inflammation resulting from dietary changes.

A third factor to consider is that both lactose intolerance and RhA have been linked to changes in body mass index (BMI). Elevated BMI is pro-inflammatory and is associated with autoimmune diseases including RhA^14^; similarly, low BMI has been observed to suppress pro-inflammatory cytokines.^15^ Mendelian randomisation (MR) evidence has associated adult-onset lactose intolerance with reduced BMI,^16 17^ and atypical lactase persistence has been associated with increased BMI.^16^ If adult-onset lactose intolerance leads to a reduction in BMI, this may reduce the risk of RhA, even if consumption of dairy products is anti-inflammatory.

Genetic measures are by definition upstream of an environmental exposure such as intake of dairy products or changes in BMI. For this reason, we employed two genetic measures—MR and a rare variant analysis—to determine whether lactose intolerance causally affects the risk of RhA. MR uses common genetic variants as natural experimental instruments, assigning participants into groups at conception based on their genetic liability to a particular exposure. These groups are compared to determine if the exposure has a causal effect on disease outcomes.^18^ The methodology relies on using single-nucleotide polymorphisms (SNPs) as instrumental variables to estimate causal effects. For MR to establish causation, three assumptions must be met: the genetic instruments should affect the exposure, not be related to confounders, and must influence the outcome only through the exposure.

**Figure 1:**
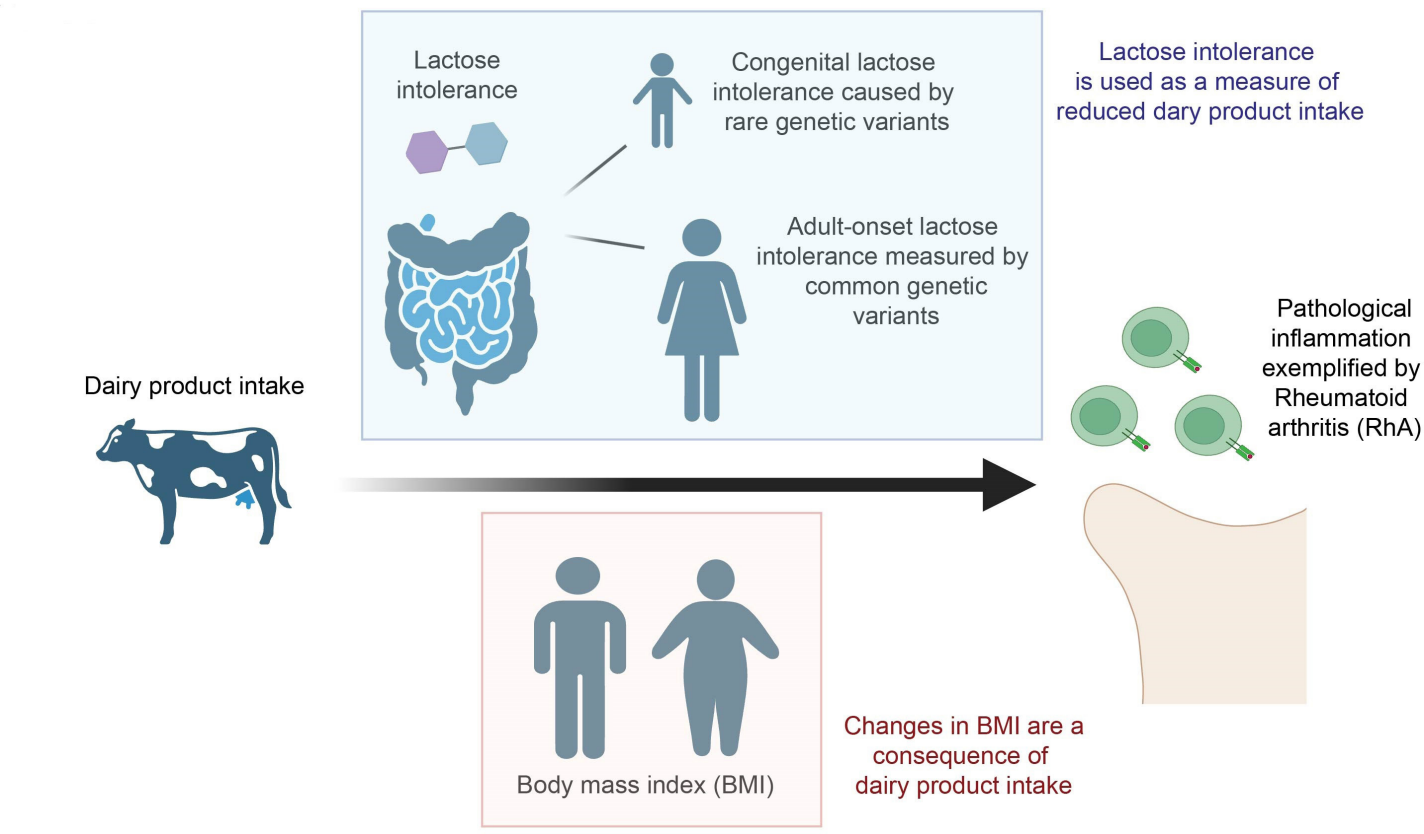
Approaches to study the effects of dairy product intake on pathological inflammation as exemplified by RhA. Dairy product intake has been proposed to both increase and decrease harmful inflammation. We have studied this problem using lactose intolerance, which necessarily reduces intake of dairy products, BMI which is pro-inflammatory but which is impacted by dairy intake, and RhA as a marker of pathological inflammation. We used genetic measures of each trait which are not subject to reverse causation or selection bias. We observed that congenital lactose intolerance is harmful, suggesting that dairy intake is anti-inflammatory. Observed protective effects of adult-onset lactose intolerance could be explained by an indirect effect of reduced BMI.

We aimed to use the comparison between congenital and adult-onset lactose intolerance and their relationship to both RhA and BMI, in order to dissect the various contributing causes to a link between dairy intake and pathological inflammation. We have defined adult-onset lactose intolerance as lactose intolerance manifesting in adulthood and likely resulting from age-related decline in lactase production.^5^ Unlike adult-onset lactose intolerance, congenital lactose intolerance is caused by rare homozygous or complex heterozygous mutations within the *LCT* gene,^4^ which encodes lactase. These mutations can be identified with high confidence, and their statistical relationship to other disease outcomes can be determined. We hypothesise that adult-onset but not congenital lactose intolerance may be confounded by an interaction with BMI which is proinflammatory. Our approach is summarised in figure 1.

## Methods

### Exposure and outcome genome-wide association studies (GWAS)

GWAS used to measure genetic liability to adult-onset lactose intolerance was obtained from the FinnGen Biobank and included 453 733 Finnish biobank donors of whom 445 reported lactose intolerance with a mean age of onset of 38.3 years. Cases were included based on hospital-recorded International Classification of Diseases (ICD)-10 codes.

GWAS used to measure genetic determinants of BMI was a meta-analysis of 125 studies performed by the GIANT consortium,^19^ which included 339 224 participants, of whom 322 154 individuals were of European descent. Some cohorts within GIANT measured BMI directly, whereas others utilised self-reported BMI, but this detail was included as a covariate in the meta-analysis.

GWAS used to measure genetic liability to osteoporosis was performed in UK Biobank participants and included 484 598 participants of European ancestry, of whom 7751 were diagnosed with osteoporosis.^20^ Here, osteoporosis was defined by quantitative bone mineral density measured via dual-energy X-ray absorptiometry at the femoral neck based on WHO T-score criteria.

GWAS used to measure RhA was a meta-analysis of UK Biobank, FinnGen and Biobank Japan cohorts including 628 000 participants.^21^ Specific to RhA, the study included 8255 European ancestry cases and 409 001 European ancestry controls; and 5348 East Asian ancestry cases and 173 268 East Asian ancestry controls. The definition of RhA in each study was slightly different: UK Biobank and FinnGen used hospital-recorded ICD-10 codes whereas Biobank Japan relied on physician-confirmed anti-cyclic citrullinated peptide-positive RhA.

GWAS used to measure serum concentration of C reactive protein (CRP) was performed in Biobank Japan^22^ participants including 10 112 participants.

### Two-sample MR

For all MR tests, we report the multiplicative random effects inverse variance weighted (IVW)^23^ estimate of causal inference because this carries the most statistical power and is more robust to heterogeneity than a fixed effects IVW.^18^ Genetic instruments were selected with a conservative p value cut-off (p<5E−8) except for lactose intolerance, where we used a positive control, the causal link between lactose intolerance and osteoporosis, to guide selection of instruments. Identified SNPs within a 10 kb window were clumped for independence using a stringent cut-off of R^2^≤0.001 within a European reference panel; where SNPs were in linkage disequilibrium (LD), those with the lowest p value were retained. Where an exposure SNP was unavailable in the outcome dataset, a proxy with a high degree of LD (R^2^≥0.9) was identified within a European reference population. The effects of SNPs on outcomes and exposures were harmonised in order to ensure that the beta values were signed with respect to the same alleles. For palindromic alleles, those with minor allele frequency (MAF) >0.42 were omitted from the analysis in order to reduce the risk of errors due to strand issues.

In order to increase confidence in the IVW results, we performed a series of robust MR measures and sensitivity analyses. We used an F-statistic to measure the strength of the association between instrumental SNPs and the exposure of interest. An F-statistic >10 indicates that an SNP-derived estimate has a bias of <10% of its intragroup variability and signifies an acceptable instrument. Pleiotropy occurs between SNPs where the difference in effect size for the exposure is not proportional to the difference in effect size for the outcome, and is usually due to a violation of one of the key assumptions underlying MR, the assumption that instrumental SNPs should be associated with the outcome only through the exposure.^18^ To account for pleiotropy, we removed SNPs where the p value for the association with the outcome was lower than for the association with the exposure of interest. As IVW estimates are vulnerable to pleiotropic SNPs, we used Cochran’s Q test (p>0.05) as a sensitivity measure to detect heterogeneity indicating pleiotropy. Moreover, radial-MR^24^ was used to remove statistically significant outlier SNPs. The I^2^ statistic was used to measure the heterogeneity between variant-specific causal estimates, with a low I^2^ indicating bias toward the null hypothesis.^25^ TwoSampleMR (version 0.5.6), MR (V.0.5.1) and RadialMR (V.1.0) R packages were used for all MR analyses.

### Multivariable MR

Multivariable MR (MVMR)^26 27^ was used to test whether the effect of adult-onset lactose intolerance on RhA was conditional on changes in BMI. GWAS summary statistics were obtained as for two-sample MR analyses. The p value cut-offs used to choose instrumental SNPs for each exposure were chosen so as to achieve adequate instrument strength for both exposures (conditional F-statistic >10 for each exposure^28^). Reported results showed no evidence of instrument heterogeneity (Cochran’s Q-test p>0.05). Exposures were derived from independent cohorts, and therefore, a correction for the covariance between the effect of the genetic variants on each exposure was not necessary. MVMR was implemented using the MVMR (V.0.3)^27^ and MendelianRandomisation (V.0.5.1)^26^ R packages.

### Rare genetic variant burden testing

To perform rare genetic variant burden testing to determine the effect of congenital lactose intolerance on risk of RhA, we used whole-genome sequencing data from UK Biobank^29^; specifically, we used UK Biobank 500 k WGS (V.2) including 484 111 genomes. We considered both juvenile arthritis and RhA because RhA in children is considered within the diagnosis of juvenile arthritis.

We considered variants to be high-quality variant calls based on coverage, mapping quality, genotype quality and Hardy-Weinberg equilibrium. We were aiming to identify individuals with a complete LOF in *LCT* and, therefore, we considered non-synonymous variants which were rare (MAF <0.0005 in both UK Biobank and GnomAD^30^) and either homozygous or complex heterozygous (ie, there were two qualifying but different variants within the same individual). Identified rare variants with a common biological effect were collapsed into a single Fisher’s exact two-sided test to determine whether the burden of variants is different in cases of inflammatory arthritis versus controls.

## Results

### Congenital lactose intolerance increases the risk of inflammatory arthritis

Congenital lactose intolerance is caused by LOF mutations within *LCT*, which encodes the lactase enzyme.^4^ The mutations occur in a homozygote or compound heterozygote pattern and compromise both missense and nonsense mutations. We applied this model in UK Biobank participants to test whether congenital lactose intolerance is causally linked to risk of RhA (Methods); whole genome sequencing data were available from >1 00 000 participants (table 1). RhA in children is considered within the diagnosis of juvenile arthritis, and therefore, we considered the association between congenital lactose intolerance and both juvenile arthritis and RhA. Using hospital-recorded ICD-10 codes as the diagnostic standard, congenital lactose intolerance is causally associated with juvenile arthritis (Fisher’s exact test, p=0.03, OR=37.6, table 1) and with adult-onset seropositive RhA (p=0.05, OR=18.8, table 1).

**Table 1:** Congenital lactose intolerance is associated with risk of inflammatory arthritis

Phenotype	Source of diagnosis	P value	Number of samples	Number of cases	Cases with congenital lactose intolerance (%)	OR (LCI, UCI)
Juvenile arthritis	Hospital records	0.03	353 932	98	1.02	37.6 (5.2, 272.3)
Juvenile arthritis	Union	0.04	118 882	153	0.65	25.2 (3.4, 185.7)
Rheumatoid arthritis	Hospital records	0.05	208 882	183	0.55	18.8 (2.6, 136.3)
Rheumatoid arthritis	Union	0.1	110 535	399	0.25	9.9 (1.3, 72.8)
Arthritis unspecified	Union	0.03	162 255	29 881	0.05	2.0 (1.1, 3.8)

Mutations within *LCT*, which encodes the lactase enzyme, were considered under a recessive model (Methods). Cases were considered to be individuals who reported either juvenile arthritis, which includes RhA in individuals aged <16 years, or seropositive adult-onset RhA. ‘Union’ refers to diagnosis recorded in any of self-reported data via questionnaire, hospital records (ICD-9/ICD-10), primary care records, or death registry data. Odd’s ratio (OR) is quoted with lower confidence interval (LCI) and upper confidence interval (UCI) to delineate the 95% CI. OR >1 indicates higher risk of arthritis in mutation carriers.

RhA, rheumatoid arthritis.

### Positive control analysis to guide genetic instrument selection for measurement of adult-onset lactose intolerance in MR

Lactose intolerance is causally related to osteoporosis^31 32^ and this relationship is biologically plausible mechanism due to the reduced calcium intake associated with reduced dairy product intake. We took advantage of this fact to derive an appropriate set of genetic instruments for inferring lactose intolerance in our subsequent MR study of RhA.

**Figure 2:**
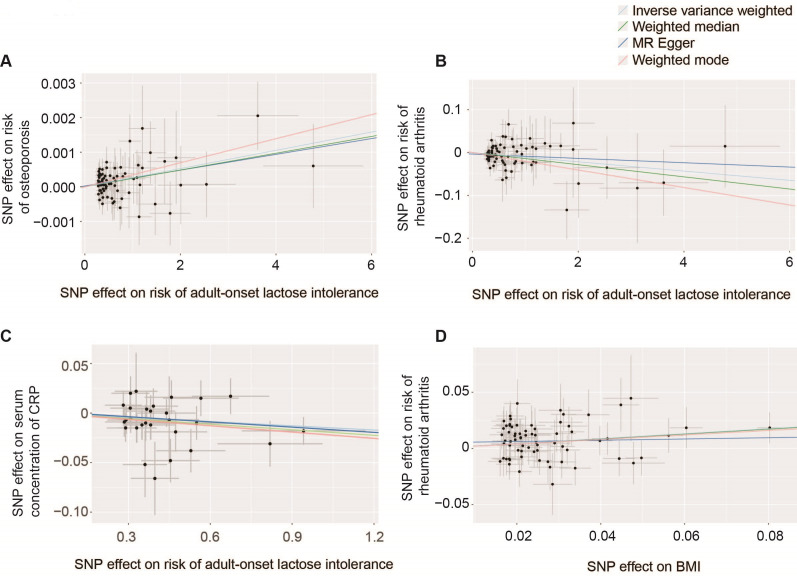
Two-sample Mendelian randomisation (MR) tests. Scatter plots demonstrate a significant positive association of adult-onset lactose intolerance with risk of osteoporosis (**A**); a significant protective effect of adult-onset lactose intolerance on risk of RhA (**B**); a negative association of adult-onset lactose intolerance with serum concentration of CRP (**C**) and a positive association of BMI with risk of RhA (**D**). Trend lines are shown for the IVW and robust MR tests. Each point represents the effect size (beta) and SEs for each SNP–outcome relationship. Positive beta indicates a positive association between the exposure and the outcome. BMI, body mass index; CRP, C reactive protein; IVW, inverse variance weighted; RhA, rheumatoid arthritis; SNP, single-nucleotide polymorphism.

The p value cut-off for choice of genetic instruments (SNPs) in MR is a compromise: When the cut-off is too low, informative instruments will be lost, but when it is too high, non-informative instruments will be introduced and instrument pleiotropy is more likely to occur.^33^ We tested multiple p value cut-offs between 5e−8 and 5e−4 in order to identify the most appropriate (online supplemental table 1). After clumping, only one SNP met a genome-wide threshold (p<5e−8) for association with lactose intolerance; this is rs182549, which is part of a recognised haplotype associated with adult-onset lactose intolerance.^34^ A single genetic instrument does not enable important quality controls including robust MR tests and sensitivity analyses (Methods). A p value cut-off of 5e−5 produced the most significant result (IVW, p=9.4e−4, beta=2.6e−4, SE=8.0e−5, table 2, figure 2A).

**Table 2:** Test statistics and sensitivity measures for MR analyses of adult-onset lactose intolerance, RhA and BMI

Test	Effect of lactose intolerance on risk of osteoporosis	Effect of BMI on risk of RhA	Effect of lactose intolerance on risk of RhA	Effect of lactose intolerance on serum CRP
P value to select SNPs	5e−5	5e−8	5e−5	5e−5
Number of SNPs	75	70	77	28
IVW P value	9.4e−4	5.6e−4	0.01	0.03
IVW beta	2.6e−4	0.22	−0.01	−0.01
Weighted median P value	0.05	0.04	0.03	0.09
Weighted median beta	2.4e−4	0.21	−0.01	−0.02
Egger P value	0.1	0.7	0.54	0.37
Egger beta	2.2e−4	0.06	−0.005	−0.02
Weighted mode P value	0.07	0.1	0.06	0.11
Weighted mode beta	3.5e−4	0.2	−0.02	−0.02
Mean F test	21.3	68.0	21.0	21.0
IVW Cochran’s *Q* test P value	0.99	0.79	0.99	0.66
Radial MR outlier SNPs	3	8	5	2
I^2^	0.95	0.98	0.95	0.95

Positive beta indicates a positive association between the exposure and the outcome.

BMI, body mass index; CRP, C reactive protein; IVW, inverse variance weighted; MR, Mendelian randomisation; RhA, rheumatoid arthritis; SNPs, single-nucleotide polymorphisms.

### Adult-onset lactose intolerance is protective against the development of RhA

Our positive control analysis of the causal relationship between adult-onset lactose intolerance and osteoporosis enabled optimum instrument selection for an MR test to determine whether adult-onset lactose intolerance is causally linked to the development of RhA. Genetic liability to adult-onset lactose intolerance is protective against the development of RhA (IVW, p=0.01, beta=−0.01, table 2, figure 2B). This result was also statistically significant in robust MR tests and, based on the sensitivity tests performed, none of these tests was invalidated by instrument pleiotropy or weak instruments (Methods, table 2). Similarly, the recognised haplotype associated with adult-onset lactose intolerance^34^ is associated with reduced risk of RhA (SAIGE, p=0.04, beta=−5e−4). An reverse-MR test to determine whether the development of RhA is causally linked to adult-onset lactose intolerance was non-significant (IVW, p=0.14), suggesting that our result stems from an effect of lactose intolerance on the risk of RhA and not from shared heritability.

We have used RhA as a proxy for harmful inflammation. CRP is a protein produced by the liver in response to inflammation, including RhA.^35^ As an additional validation, we used the same set of instruments to determine whether adult-onset lactose intolerance is causally associated with the serum concentration of CRP. Consistent with our analysis of RhA, we discovered a negative association between genetic liability to adult-onset lactose intolerance and serum CRP (IVW, p=0.03, beta=−0.01, table 2, figure 2C). There was no evidence for the reverse association (IVW, p=0.58).

### Protective effect of adult-onset lactose intolerance against the development of RhA is conditional on elevated BMI

BMI is pro-inflammatory and is associated with autoimmune diseases including RhA.^14^ Given that reduced BMI is associated with adult-onset lactose intolerance,^16 17^ we hypothesised that the protective effect of adult-onset lactose intolerance on the development of RhA might be mediated via reduced BMI.

First, we sort to confirm the causal relationship between elevated BMI and risk of RhA. Using a p value cut-off of 5e−8 to select instruments, genetic liability to higher BMI has a significant causal effect on the risk of RhA (IVW, p=5.9e−7, beta=0.24, SE=0.05, table 2, figure 2D). This result was also statistically significant in robust MR tests and, based on the sensitivity tests performed, none of these tests was invalidated by instrument pleiotropy or weak instruments (Methods, table 2).

The protective effect of adult-onset lactose intolerance was non-significant when conditioned on BMI (adult-onset lactose intolerance p=0.36, beta=−0.002, SE=0.003 and BMI p=0.04, beta=0.14, SE=0.07). The MVMR analysis achieved adequate instrument strength for both exposures, and there was no evidence of instrument heterogeneity (Methods).

## Discussion

Dairy products form a significant portion of the human diet almost universally. Therefore, it is important that the health benefits or harms of consumption of dairy products are well understood. Unfortunately, there has been significant controversy in the literature leading to conflicting advice with respect to the role of dairy intake in terms of systemic inflammatory response. Here, we have used traits linked to dairy consumption—lactose intolerance and BMI—to dissect the role of dairy consumption on harmful inflammation as exemplified by RhA and serum concentration of CRP. We have confined our analysis to genetic measures which are fixed at conception, and therefore, our results are less vulnerable to selection bias or reverse causation than a conventional observational study. Moreover, this approach enables us to take advantage of several large datasets including UK Biobank, FinnGen Biobank and Biobank of Japan.

We have demonstrated that congenital and adult-onset lactose intolerance have opposite effects on the risk of RhA. Congenital lactose intolerance is causally linked to the risk of RhA, which is consistent with the idea that dairy consumption is anti-inflammatory. This is supported by a recent meta-analysis,^7^ and with the observation that dairy products contain a number of anti-inflammatory lipids.^8^ Conversely, adult-onset lactose intolerance has a protective effect and reduces the risk of RhA. We have provided evidence to explain this apparently contradictory observation, by demonstrating that the protective effect of adult-onset lactose intolerance is conditional on reduced BMI. Indeed, after controlling for BMI, the protective effect of adult-onset lactose intolerance becomes non-significant. Similarly, there is evidence from observational studies of a significant *inverse* relationship between dairy intake and BMI after controlling for physical exercise and total dietary energy intake.^36^ This suggests that dairy product intake in moderation does not inevitably lead to harmful increases in BMI and should, in light of our data, be encouraged for its positive effect on risk of autoimmune disease.

We did not control for BMI in our analysis of congenital lactose intolerance because of the technical challenge of combining common and rare variant analyses. However dairy intake in babies and children has been specifically associated with higher BMI.^37^ This suggests that congenital lactose intolerance may actually be protective against obesity, which makes the positive association we observe between lifelong dairy avoidance and RhA more striking. A limitation of our analysis of congenital lactose intolerance is the small numbers of diseased patients identified who have LOF mutations within both *LCT* alleles.

An alternative explanation for the difference between our observations regarding congenital and adult-onset lactose intolerance is that dairy products have a different effect in development^38^ compared with in adulthood. Congenital lactose intolerance may, therefore, increase the risk of RhA via a mechanism which is not affected by adult-onset lactose intolerance where onset occurs after development has completed. It was not possible to discount this possibility in our analysis; however, we can say that we do not find any evidence for a pro-inflammatory effect of dairy products, given that BMI completely explains the protective effect of adult-onset lactose intolerance.

A limitation of our study is that there is sample overlap between the GWAS used to measure lactose intolerance and the GWAS used to measure liability to RhA; the lactose intolerance GWAS was performed in FinnGen which forms a subset of the GWAS used for RhA. If weak instruments are present, this can produce a biased result.^39^ We have countered this by performing a positive control analysis to guide instrument selection for the measurement of lactose intolerance, and we showed that as measured by the F-statistic, we achieved adequate instrument strength.

Another limitation of our study is that the genetic measures we have employed do not differentiate between different dairy foods which are likely to contain varying proportions of anti-inflammatory lipids.^8^ Indeed, which specific component of dairy products is responsible for changes in propensity to harmful inflammation is unknown but is of key importance for translation of our findings into benefit for human health.

In summary, in this study, we have presented evidence that avoidance of dairy product consumption can increase the risk of RhA. Our findings may extend to other diseases underpinned by systemic inflammatory processes, and consequently, this study supports the public health recommendations that dairy should form part of a healthy, balanced diet. Future research should focus on the identification of which components of dairy products are anti-inflammatory and whether supplements could be used to mitigate the harmful effects of congenital lactose intolerance.

## Supplementary material

10.1136/bmjnph-2024-001036 online supplemental file 1

## Data Availability

No data are available.
